# Complexity in Estimation of Esomeprazole and its Related Impurities’ Stability in Various Stress Conditions in Low-Dose Aspirin and Esomeprazole Magnesium Capsules

**DOI:** 10.3797/scipharm.1212-13

**Published:** 2013-02-18

**Authors:** Palavai Sripal Reddy, Kishore Kumar Hotha, Shakil Sait

**Affiliations:** 1 Analytical Research and Development, IPDO, Dr. Reddy’s. Ltd. Hyderabad, 500072, India.; 2 JNT University, Kukatpally, Hyderabad, 500085, A.P, India.

**Keywords:** RP-HPLC, Forced degradation, Validation, Esomeprazole, Aspirin, Acetylsalicylic acid, Method development, Method validation, Degradants, Mmigration

## Abstract

A complex, sensitive, and precise high-performance liquid chromatographic method for the profiling of impurities of esomeprazole in low-dose aspirin and esomeprazole capsules has been developed, validated, and used for the determination of impurities in pharmaceutical products. Esomeprazole and its related impurities’ development in the presence of aspirin was traditionally difficult due to aspirin’s sensitivity to basic conditions and esomeprazole’s sensitivity to acidic conditions. When aspirin is under basic, humid, and extreme temperature conditions, it produces salicylic acid and acetic acid moieties. These two byproducts create an acidic environment for the esomeprazole. Due to the volatility and migration phenomenon of the produced acetic acid and salicylic acid from aspirin in the capsule dosage form, esomeprazole’s purity, stability, and quantification are affected. The objective of the present research work was to develop a gradient reversed-phase liquid chromatographic method to separate all the degradation products and process-related impurities from the main peak. The impurities were well-separated on a RP8 column (150 mm × 4.6mm, X-terra, RP8, 3.5μm) by the gradient program using a glycine buffer (0.08 M, pH adjusted to 9.0 with 50% NaOH), acetonitrile, and methanol at a flow rate of 1.0 mL min^−1^ with detection wavelength at 305 nm and column temperature at 30°C. The developed method was found to be specific, precise, linear, accurate, rugged, and robust. LOQ values for all of the known impurities were below reporting thresholds. The drug was subjected to stress conditions of hydrolysis, oxidation, photolysis, and thermal degradation in the presence of aspirin. The developed RP-HPLC method was validated according to the present ICH guidelines for specificity, linearity, accuracy, precision, limit of detection, limit of quantification, ruggedness, and robustness.

## Introduction

**Fig. 1:**
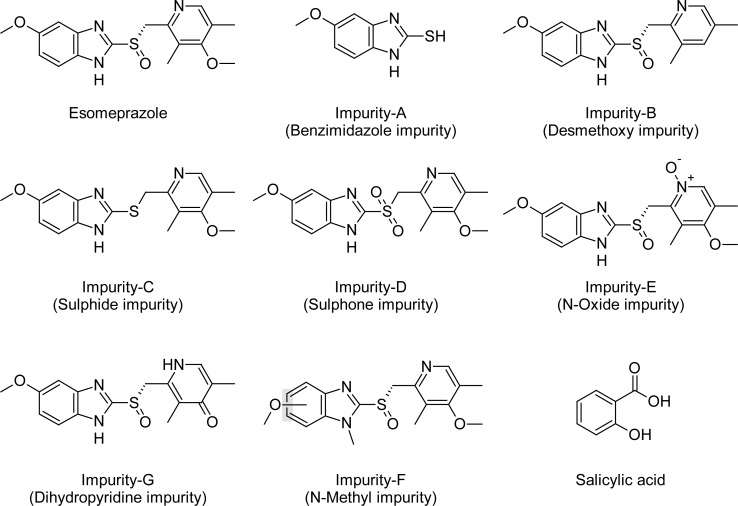
Chemical Structures of Esomeprazole and its Related impurities

Esomeprazole (Figure 1) is a proton pump inhibitor used in the treatment of dyspepsia, peptic ulcer disease (PUD), gastroesophageal reflux disease (GORD/GERD), and Zollinger-Ellison syndrome. Esomeprazole, the S-enantiomer of omeprazole, shows improved efficacy of this single enantiomer product over the racemic mixture of omeprazole. Esomeprazole is a proton pump inhibitor, which reduces acid secretion through the inhibition of ATPase in gastric parietal cells, by inhibiting the functioning of this enzyme, so the drug prevents formation of gastric acid. The primary uses of esomeprazole are for gastroesophageal reflux disease, treatment of duodenal ulcers caused by H. pylori, prevention of gastric ulcers in those on chronic NSAID therapy, and treatment of gastrointestinal ulcers associated with Crohn’s disease [1–3]. *In vivo* investigations demonstrated that ESO is chirally stable after administration. ESO is 97% bound to plasma proteins. Omeprazole is a racemic composition of its two optical isomers, S-omeprazole (esomeprazole) and R-omeprazole, which have demonstrated stereo-selective metabolisms [4–6]. Aspirin (ASP), also known as acetylsalicylic acid, is a salicylate drug, often used as an analgesic to relieve minor aches and pains, as an antipyretic to reduce fever, and as an anti-inflammatory medication. Aspirin, by irreversibly acetylating cyclo-oxygenase (COX), reduces the production of thromboxane A2 (TXA2) in platelets and prevents platelet aggregation [7]. Aspirin can also reduce prostacyclin (PGI2) production in endothelial cells and cause vasoconstriction. One of the side effects associated with the use of aspirin is gastrointestinal ulcers. Aspirin has a long history of therapeutic use, not only for its analgesic, antipyretic, and anti-inflammatory properties, but also for its anti-thrombotic properties, which are of value in states of platelet hyperaggregability. Aspirin binds irreversibly to the enzyme cyclo-oxygenase-1 (COX-1) in platelets, leading to its antiplatelet effect [8]. Side effects of aspirin treatment are mainly dyspeptic symptoms, gastrointestinal (GI) lesions, and increased gastrointestinal and overall bleeding, which are consequences of the blockage of prostaglandin synthesis through inhibition of various COX enzymes. This leads to a decrease in mucosal protection, which in turn predisposes the patient to mucosal lesions such as peptic ulcers and peptic ulcer bleeding. Esomeprazole is a proton pump inhibitor (PPI) which is indicated, amongst other indications, for the prevention of gastric and duodenal ulcers associated with NSAID therapy (including aspirin therapy). There are many drug products containing aspirin at 100 mg strength as enteric-coated tablets. These are the only low-dose aspirin monotherapy drug products apart from breaking a 300 mg tablet in half, which is probably done by a small proportion of patients taking low-dose aspirin for cardiovascular protection [9–11].

A combination of the esomeprazole and aspirin assay/related impurities method was traditionally difficult due to these drugs’ stability in aqueous solutions, dosage variations, and their absorption differences in the UV region. The literature reveals that aspirin is stable in acidic form, whereas esomeprazole is stabile in basic solutions. The objective of the present research work is to establish the specificity and stability of esomeprazole in the presence of aspirin and its related impurities, which gives a precise and accurate quantification of esomeprazole in the pharmaceutical dosage forms of esomeprazole and aspirin. Several HPLC [12–16] and LC-MS/MS [17–29] methods have been reported for the estimation of omeprazole alone or along with its metabolites and in some combination with NSAID in various pharmaceutical dosage forms and in biological fluids. The current research article provides a fully validated and stability-indicating method as per the ICH guidelines and its degradation behavior in the presence of aspirin and its potential metabolite salicylic acid. The present research article successfully measured the migration content of salicylic acid in the combined capsule dosage form and showed a reliable, accurate quantification of esomeprazole and its related impurities in the presence of aspirin and its potential degradants.

## Materials and Methods

### Chemicals and reagents

Samples of esomeprazole, aspirin and its impurities’ standards with purities of 99.8% were supplied from Dr. Reddy’s laboratories Ltd, Hyderabad, India (Figure 1). The HPLC grade acetonitrile, ethanol, methanol, analytical grade glycine, triethylamine, sodium hydroxide, disodium tetraborate decahydrate, and edetate disodium were purchased from Merck, Darmstadt, Germany. High-purity water was prepared by using the Millipore Milli-Q water purification system.

### Equipment

The HPLC system, used for method development, forced degradation studies, and method validation, was a Waters HPLC system equipped with a photodiode array detector, from Waters Corp. (Milford, MA, USA). The output signal was monitored and processed using Empower software (Waters). A water bath equipped with a temperature controller was used to carry out the degradation studies for all solutions. Photostability studies were carried out in a photostability chamber and thermal stability studies were performed in a dry air oven (Mack Phar-ManTech, Hyderabad, India).

### Chromatographic Conditions

The chromatographic column used was a Waters X-Terra RP 8 Column 150 mm×4.6 mm, 3.5 μm, all obtained from Waters Corp. (Milford, MA, USA). The gradient LC method consisted of a glycine (0.08M) buffer pH 9.0 as mobile phase-A, and acetonitrile and methanol in a ratio of 85/15v/v as mobile phase-B. The flow rate of the mobile phase was 1.0 ml/min with a gradient programme of 0.001/06, 12/12, 17/18,17.5/20, 30/40, 40/46, 43/85, 45/06, 50/06. The column temperature was maintained at 30°C and the detection was monitored at a wavelength of 305nm. The injection volume was 20μl. The diluent used was in a ratio of 80:20 (7.6g of disodium tetraborate and 1.0 g of edetate disodium in 1 liter of Milli-Q water, pH-adjusted to 11.0 with a 50 % sodium hydroxide solution): ethanol.

### Preparation of Solutions

The diluent used for the standard and sample preparation was a mixture of ethanol and disodium tetraborate buffer in the ratio of 80:20 (v/v).

A standard solution of esomeprazole (0.8 ppm), salicylic acid (0.6ppm), and test preparation (400 ppm) was prepared by dissolving an appropriate amount in the diluent.

### Specificity

Specificity is the ability of the method to measure the analyte response in the presence of its potential impurities [30–32]. Stress testing of the drug impurities can help identify the likely degradation products, which can in turn help establish the degradation pathways and the intrinsic stability of the molecule, and validate the stability-indicating power of the analytical procedures used.

The specificity of the developed LC method for esomeprazole was determined in the presence of its related impurities and in the presence of salicylic acid. Forced degradation studies were also performed on esomeprazole to provide an indication of the stability-indicating property and specificity of the proposed method. The stress conditions employed for the degradation study included light (carried out as per ICH Q1B), dry heating done at 105°C for about 2 hrs., acid hydrolysis (refluxed with 0.1N HCl solution for about 120minutes at 60°C), base hydrolysis (refluxed with 0.1N NaOH solution for about 120 minutes at 60°C), water hydrolysis, and oxidation (treated with 3% hydrogen peroxide (H_2_O_2_) for about 120 minutes at RT). Sunlight, thermal, and UV degradation were also performed and the purity of the stressed samples was checked by using a photodiode array detector (PDA). The purity factor was within the threshold limit obtained in all stressed samples, which demonstrates analyte peak homogeneity. The specificity of esomeprazole was shown by spiking all esomeprazole and its related impurities at the specification level (i.e. 0.5% of analyte concentration, which is 0.2 mg/mL).

### Analytical method validation

The developed chromatographic method was validated for linearity, precision, accuracy, sensitivity, robustness, and system suitability as per ICH guidelines [31–33].

#### Precision

The precision of the test method was evaluated by analyzing six test samples of low-dose aspirin and esomeprazole capsules. It was spiked with its seven impurities and with the salicylic acid impurity at 0.5%, and then analyzed. The % RSD of the area of each impurity was calculated.

#### Limit of detection (LOD) and limit of quantification (LOQ)

The limit of detection and limit of quantification were established based on the signal-to-noise ratio. A series of solutions having esomeprazole and its related impurities were injected. The limit of detection for the impurity was established by identifying the concentration which gives a signal-to-noise ratio of about 3. The limit of quantification was established by identifying the concentration which gives a signal-to-noise ratio of about 10. The precision of esomeprazole impurities at about the limit of quantification level was conducted. Six test preparations having impurities at the level of about the limit of quantification were prepared and injected into the HPLC system.

#### Linearity and Range

The linearity for esomeprazole and its related impurities was prepared from the limit of quantification level to 150% of the target concentration (0.5%). The linearity was established for salicylic acid from the limit of quantification level to 150% of the target concentration (0.3%) and injected into the HPLC system.

#### Accuracy

The accuracy of esomeprazole, its related impurities, and salicylic acid was prepared at about the limit of quantification to 150% of the target concentration level. Test solutions spiked with esomeprazole impurities at about the limit of quantification to 150% of the target concentration were prepared in triplicate and injected into HPLC system.

#### Robustness

To determine the robustness of the developed method, experimental conditions were deliberately changed and the resolution (*Rs*) between esomeprazole and its impurity was evaluated. The flow rate of the mobile phase was 1.0 mL·min^−1^. To study the effect of flow rate on the developed method, 0.2 units of flow was changed (*i.e*. 0.8 and 1.2 mL·min^−1^).

The effect of column temperature on the developed method was studied at 25°C and 35°C instead of 30°C. In all of the above-varied conditions, the components of the mobile phase were held constant, and it was established that the allowable variation in pH of the buffer in the mobile phase was from pH 8.8 to pH 9.2 instead of 9.0.

#### Solution Stability and Mobile Phase Stability

The solution stability of esomeprazole, its related impurities, and salicylic acid was carried out by leaving both spiked samples and the unspiked sample solution in a tightly capped volumetric flask at room temperature for 10 hrs and then in a refrigerator for 48 hrs. Content impurity was determined at every two hr interval, up to the study period. Mobile phase stability was also carried out for 48 hrs by injecting the freshly prepared sample solutions at every six hr interval. Esomeprazole-related impurities and the salicylic acid impurity were checked in the test solutions. The mobile phase preparation was kept constant during the study period.

## Results and Discussion

### Method Development and Optimization

The main complexity of the present research work was to develop a stability-indicating method for the estimation of Esomeprazole in low-dose aspirin and esomeprazole pharmaceutical dosage forms in the presence of aspirin. Challenges were observed in the selection of the stationary phase and mobile phase due to the migration of an impurity that was observed in esomeprazole as salicylic acid, produced from aspirin in the capsules of low-dose aspirin and esomeprazole. The main target of the chromatographic method was to get the separation of critically close eluting degradable peaks from main peak and to know the effect of salicylic acid that was produced in the sample preparation and its interference with esomeprazole. Impurities were co-eluted by using different stationary phases like C18, phenyl, and cyano, and different mobile phases containing buffers like phosphate, sulphate, and acetate with different pH’s (7–9), and using organic modifiers like acetonitrile, methanol, and ethanol in the mobile phase. After several scientific trials and optimization of the stationary phase, column temperature, flow rate, mobile phase, and pH, the chromatographic separation was achieved on the X-Terra RP-8 150 × 4.6mm 3.5 μm column. The X-terra RP8 column has good selectivity and ruggedness in higher pH’s. A 3.5 μ submicron column gave better separation between the seven impurities of esomeprazole, with proper retention and peak shape of the salicylic acid in higher pH buffer conditions. The gradient LC method consisted of a buffer as mobile phase-A, acetonitrile, and methanol in the ratio of 85/15v/v as mobile phase-B. The buffer solution contained 6gms of glycine in 1 liter of Milli-Q water, pH-adjusted to 9.0 ± 0.1 with a 50% sodium hydroxide solution (buffer). The glycine buffer gave better selectivity and robust mobile phase stability at a higher pH, with a sodium borate and EDTA combination providing a basic environment to protect against further degradation of the esomeprazole in the presence of the salicylic acid produced from aspirin in the capsules.

The flow rate of the mobile phase was 1.0ml/min. gradient programme Time/%v mobile phase-B 0.001/06,12/12,17/18,17.5/20,30/40,40/46,43/85,45/06,50/06. The temperature of the column was maintained at 30°C and the detection was monitored at a wavelength of 305nm. The injection volume was 20μl. The diluents used as the buffer [7.6g of disodium tetraborate and 1.0 g of edetate disodium in 1 liter of Milli-Q water pH-adjusted to 11.0 ± 0.1 with 50% sodium hydroxide solution]:ethanol in the ratio of (80:20)]. The concentration was 0.2 mg·mL^−1^ for the related impurities method. The peak shape of esomeprazole was found to be symmetrical. In the optimized conditions esomeprazole, its related impurities, and the salicylic acid impurity were well-separated with a resolution of greater than 2.5.

**Fig. 2:**
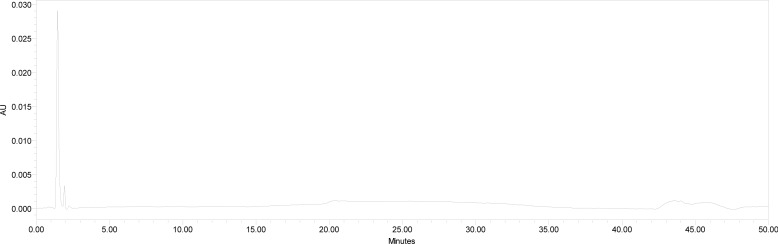
Typical HPLC Chromatogram of Diluent

**Fig. 3:**
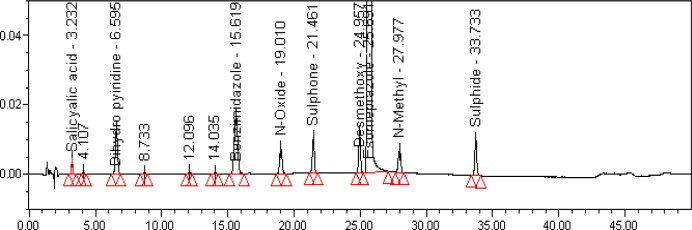
Typical HPLC Chromatogram of Impurities Blend Solution

**Fig. 4:**
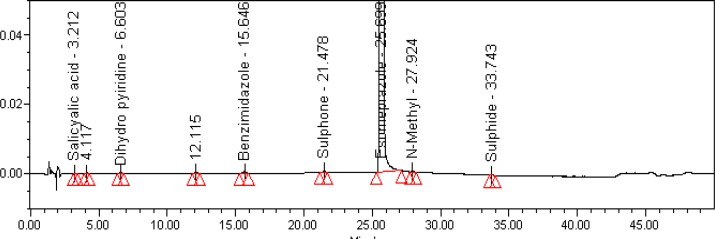
Typical HPLC Chromatogram of Sample

The relative response factor for all of the mentioned impurities against the esomeprazole was established. Results are given in Table 6, the system suitability results are given in Table 1, the developed HPLC method was found to be specific for esomeprazole, its impurities, and salicylic acid. Figure 2, Figure 3, and Figure 4 show the chromatograms of the diluent, impurity blend solution, and the test sample solution.

### Results of Forced Degradation Studies

**Fig. 5:**
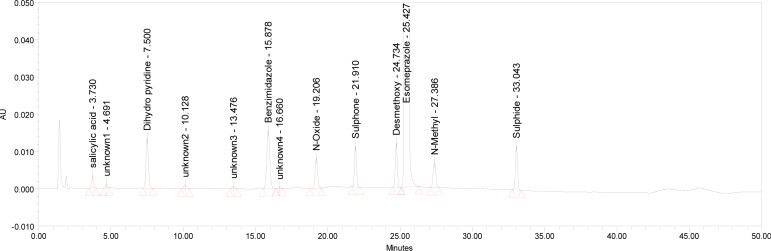
Typical HPLC Chromatogram of Acid Degradation

The drug was exposed to 0.1N HCl at 60°C for 120 min. Esomeprazole has shown significant sensitivity towards the treatment of 0.1N HCl. The drug gradually underwent degradation with time in 0.1N HCl and prominent degradation was observed (∼2%). The representative chromatogram is presented in Figure 5.

#### Degradation in Basic Solution

**Fig. 6:**
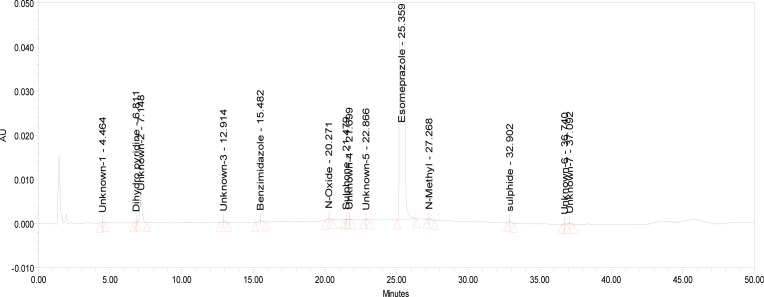
Typical HPLC Chromatogram of Base Degradation

The drug was exposed to 0.1N NaOH at 60°C for 120 min. Esomeprazole has shown mild sensitivity towards the treatment of 0.1N NaOH. The drug gradually underwent degradation with time in 0.1N NaOH and degradation was observed (∼2.5%). The representative chromatogram is presented in Figure 6.

#### Oxidative Conditions

**Fig. 7:**
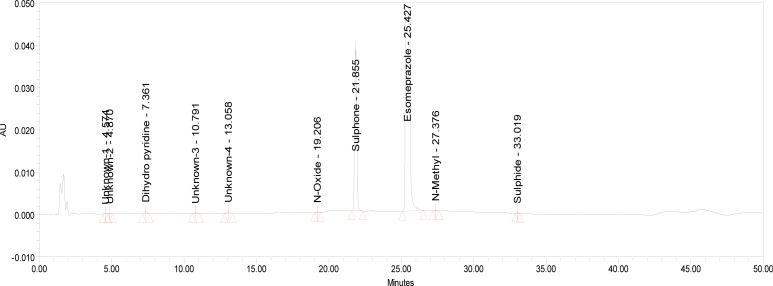
Typical HPLC Chromatogram of Oxidation Degradation

**Fig. 8:**
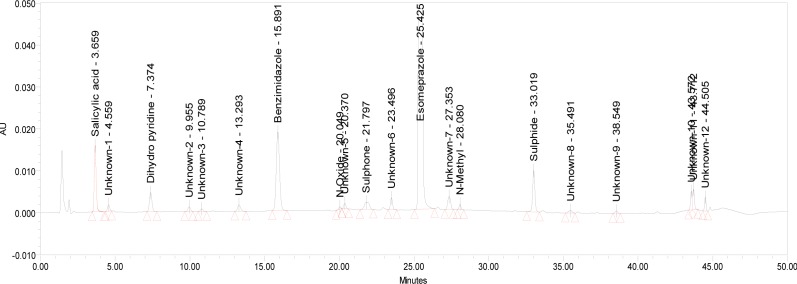
Typical HPLC Chromatogram Thermal Degradation

**Fig. 9:**
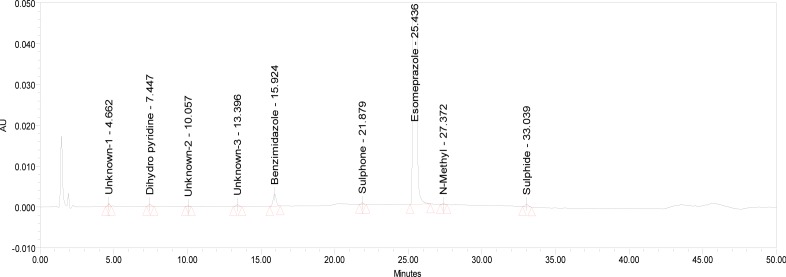
Typical HPLC Chromatogram of Sunlight Stressed Degradation

The drug was exposed to 3% hydrogen peroxide at room temperature for 120 min. Esomeprazole has shown no significant sensitivity towards the treatment of 3% hydrogen peroxide and the drug showed mild sensitivity in oxidative conditions (∼4%). Esomeprazole has shown mild degradation under forced photo and sunlight degradation. From the degradation studies, the peak purity test results derived from the PDA detector confirmed that the esomeprazole peak was homogeneous and pure in all the analyzed stress samples. The mass balance of the stressed samples was close to 99.5%. After exposing esomeprazole to sunlight (1.2 million Lux hours) and UV light (200 wt hours per sq meter), 0.55 and 1.32% degradation was observed. After dry heating at 105°C for 2 hours it was exposed to humidity at 25°C/90% RH for about 7 days. The forced degradation study results are given in Table 2. The representative chromatogram is presented in Fig. 7–9.

### Method validation

#### Accuracy

The test method was found to be accurate from the limit of quantification level to 150% of the target concentration (the target concentration is 0.5% for all of the esomeprazole impurities, for salicylic acid 0.30%, and esomeprazole of the target concentration 0.2 % of the level of unknown impurities (on placebo)). The % individual recovery at all of the spike levels of all impurities and esomeprazole was found to be within the limit. The results are summarized in Table 3.

#### Precision

The precision of the test method by injecting six samples was prepared by spiking the test preparation with the esomeprazole impurities blend solution to get Impurity-A (Benzimidazole impurity), Impurity-B (Desmethoxy impurity), Impurity-C (Sulphide impurity), Impurity-D (Sulphone impurity), Impurity-E (N-Oxide impurity), Impurity-F (N-Methyl impurity), Impurity-G (Dihydropyridine impurity) at 0.50% and salicylic acid at 0.3%. The relative standard deviation of % impurities were calculated. The experimental data is represented in Table 4.

#### Sensitivity

The limit of detection of esomeprazole and its related impurities, and salicylic acid (of analyte concentration, i.e. 0.2 mg/ml) was a 20μl injection volume. The limit of quantification of esomeprazole and its related impurities, and salicylic acid (of analyte concentration, i.e. 0.2 mg/mL) was a 20μl injection volume. The precision at the LOQ concentration were below 10 %. Experimental data is shown in Table 5.

#### Linearity and Range

The linearity was established by plotting a graph between the concentration versus peak area response of the esomeprazole and its impurities, and the salicylic acid impurity. A series of solutions of esomeprazole and its impurities with concentrations ranging from the limit of quantification level to 200% of the target concentration (0.5%) were prepared and injected into the HPLC system.

**Fig. 10:**
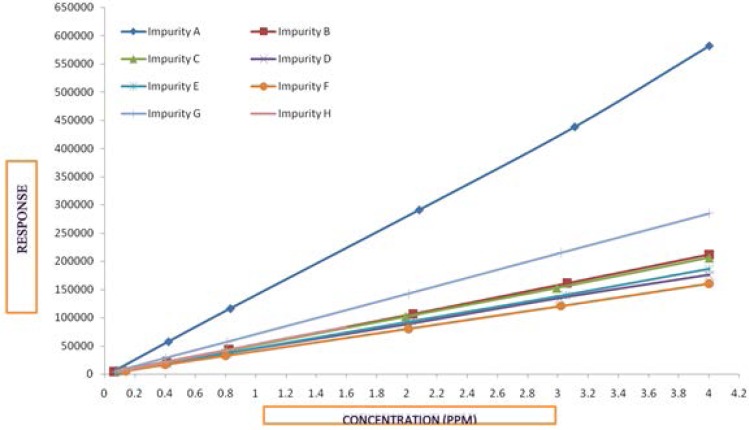
Linearity plot of Esomeprazole and its Related impurities

The detector response was found to be linear with a correlation coefficient of at least 0.997 for all esomeprazole impurities and the esomeprazole peak. The results and linearity graphs are summarized in Table 5, Table 6, Table 7, and Figure 10.

#### Robustness

Close observation of analysis results for the deliberately changed chromatographic conditions (flow rate, pH, and column temperature) revealed that the resolution between closely eluting peaks, namely esomeprazole and its related impurity, was always greater than 2.5, illustrating the robustness of the method.

#### Solution Stability and Mobile Phase Stability

No significant changes were observed in the content of esomeprazole and its related impurities, and the salicylic acid impurity during the solution stability and mobile phase stability experiments for the related impurities. The %RSD of impurities of esomeprazole during the solution stability and mobile phase stability experiments was within 0.02%. The solution stability and mobile phase stability experiment data confirm that the sample solutions and mobile phase used during related impurities determination were stable up to the study period of 10 hours at the benchtop and 48 hours in the refrigerator.

#### Related substance Analysis

An analysis was performed for the different batches of esomeprazole in the low-dose aspin and esomeprazole dosage forms in (*n* = 3) impurities ranging from 0.05%–0.16%.

## Conclusion

In this present research article, the complete degradation stress study reported for esomeprazole and its related impurities in presence of aspirin. The RP-HPLC method developed for the related impurities was linear, precise, accurate, and specific. The method was completely validated showing satisfactory data for all of the method validation parameters tested as per ICH guidelines. The developed method is stability-indicating and can be used for the routine analysis of production samples and to check the stability of esomeprazole. To the best of our knowledge, the specified method presented in the article successfully measures esomeprazole and its related impurities, and the migration impurity salicylic acid (aspirin degradant).

## Figures and Tables

**Tab. 1.** System suitability Report

**System suitability parameters**	**Observed value**	**Acceptance limit**
**From System suitability solution**		

The resolution between Impurity-B (Desmethoxy impurity) and Esomeprazole peaks.	2.8	NLT 1.5

**From Standard preparation**		

The ratio for the Esomeprazole peak areas of two standard injections	1.0	Between 0.9 to 1.1
The ratio for the Salicylic acid peak areas of two standard injections	1.0	Between 0.9 to 1.1
The Tailing factor for Esomeprazole peak in Standard	1.0	NMT 2.0
The Tailing factor for Salicylic acid peak in Standard	1.0	NMT 2.0

**Tab. 2.** Summary of Forced Degradation Study

**Stress Condition**	**Drug Product**			

	**% degradation**	**Purity angle**	**Purity threshold**	**Purity flag**
Refluxed with 10 mL of 0.1 N HCl solution for about 2 hrs at 60°C and neutralized with 0.1N NaOH	1.58	0.164	0.305	No
Refluxed with 10 mL of 0.1 N NaOH solution for about 2 hrs at 60°C and neutralized with 0.1N HCl	2.28	0.156	0.294	No
Treated with 5 mL of 3% Hydrogen peroxide (H_2_O_2_) for about 2 hrs at room temperature	3.68	0.166	0.422	No
Refluxed with purified water for about 2 hrs at 60°C	1.72	0.161	0.302	No
Exposed to Sunlight for about 1.2 Million Lux hours.	0.55	0.151	0.484	No
Exposed to UV light both at shorter and longer wavelengths for about 200 watt hours / square meter.	1.32	0.175	0.460	No
Dry heating done at 105° C for about 2.5hrs.	10.41	0.178	0.315	No
Exposed to humidity at 25°C, 90% RH for about 8 days	0.04	0.183	0.444	No

**Tab. 3.** Accuracy Data of Esomeprazole and its impurities

**Impurity-A (Benzimidazole impurity)**				**Impurity-C (Sulphide impurity)**			

20%	0.4151	0.4237	102.07	20%	0.4092	0.38503	94.07
40%	0.8303	0.83257	100.27	40%	0.8183	0.8147	99.57
100%	2.0757	2.23443	107.63	100%	2.0458	2.02453	99.80
125%	2.5946	2.9421	113.40	125%	2.5573	2.51397	98.30
150%	3.1135	3.4253	110.00	150%	3.0688	3.0063	97.90

**Impurity-B (Desmethoxy impurity)**				**Impurity-D (Sulphone impurity)**			

20%	0.3882	0.42667	109.87	20%	0.4011	0.41477	103.40
40%	0.7765	0.8162	105.13	40%	0.8023	0.81767	101.90
100%	1.9412	1.9817	102.10	100%	2.0037	1.93713	96.57
125%	2.4265	2.4069	99.20	125%	2.5071	2.46487	98.33
150%	2.9118	2.93317	100.73	150%	3.0688	2.92577	97.90

**Impurity-E (N-Oxide impurity)**				**Impurity-F (N-methyl impurity)**			

20%	0.4262	0.47127	110.23	20%	0.4232	0.4103	96.97
40%	0.8565	0.84143	98.23	40%	0.8465	0.8563	101.17
100%	2.1412	2.06797	96.60	100%	2.1161	2.12	100.20
125%	2.6765	2.58827	96.70	125%	2.6452	2.64627	100.03
150%	3.2118	3.09077	96.23	150%	3.1742	3.1815	100.23

**Impurity-G (Dihydropyridine imp.)**				**Salicylic acid impurity**			

20%	0.4029	0.4118	102.20	20%	2.5171	2.69533	107.07
40%	0.8058	0.8147	101.10	40%	5.0343	5.5512	110.27
100%	2.0146	2.14973	106.73	100%	12.0823	12.6783	104.93
125%	2.5183	2.50357	99.43	125%	15.1028	15.6488	103.60
150%	3.0219	3.06847	101.50	150%	18.1234	19.1186	105.47

**Esomeprazole Impurity**							

20%	0.1652	0.16653	100.80				
40%	0.3303	0.34637	104.90				
100%	0.8114	0.87267	107.57				
125%	1.0142	1.08993	107.47				
150%	1.2171	1.3484	110.80				


**Tab. 4.** Precision Data of Esomeprazole and its impurities

**Sample No.**	**Esomeprazole Magnesium Impurities**							

	**Impurity-A (Benzimidazole impurity)**		**Impurity-B (Desmethoxy impurity)**		**Impurity-C (Sulphide impurity)**		**Impurity-D (Sulphone impurity)**	

	**RRT**	**% Imp.**	**RRT**	**% Imp.**	**RRT**	**% Imp.**	**RRT**	**% Imp.**
1	0.61	0.456	0.97	0.457	1.31	0.475	0.84	0.486
2	0.61	0.456	0.97	0.459	1.31	0.477	0.84	0.483
3	0.61	0.453	0.97	0.46	1.31	0.47	0.84	0.486
4	0.61	0.454	0.97	0.458	1.31	0.48	0.84	0.485
5	0.61	0.449	0.97	0.458	1.31	0.473	0.84	0.483
6	0.61	0.45	0.97	0.461	1.31	0.477	0.84	0.486
AVG	–	0.453	–	0.459	–	0.475	–	0.485
%RSD	–	0.7	–	0.3	–	0.7	–	0.3

**Esomeprazole Magnesium Impurities**								

	**Impurity-E (N-Oxide impurity)**		**Impurity-F (N-Methyl impurity)**		**Impurity-G (Dihydropyridine impurity)**		**Salicylic acid impurity**	

	**RRT**	**% Imp.**	**RRT**	**% Imp.**	**RRT**	**% Imp.**	**RRT**	**% Imp.**

1	0.74	0.464	1.09	0.492	0.26	0.471	0.13	0.27
2	0.74	0.46	1.09	0.491	0.26	0.469	0.13	0.263
3	0.74	0.458	1.09	0.489	0.26	0.464	0.13	0.268
4	0.74	0.462	1.09	0.495	0.26	0.472	0.13	0.271
5	0.74	0.46	1.1	0.489	0.26	0.468	0.13	0.27
6	0.74	0.459	1.1	0.489	0.26	0.472	0.13	0.266
AVG	–	0.461	–	0.491	–	0.469	–	0.268
%RSD	–	0.5	–	0.5	–	0.7	–	1.1

**Tab. 5.** Limit of Detection and Limit of Quantification of Esomeprazole and its impurities

**Name**	**% impurity**		**Signal to Noise Ratio**	

	**LOD**	**LOD**	**LOD**	**LOD**

Impurity-A (Benzimidazole impurity)	0.003	0.009	2.7	10.4
Impurity-B (Desmethoxy impurity)	0.005	0.016	2.9	10.3
Impurity-C (Sulphide impurity)	0.01	0.023	3	10.2
Impurity-D (Sulphone impurity)	0.008	0.016	2.7	9.6
Impurity-E (N-Oxide impurity)	0.007	0.027	3	10.2
Impurity-F (N-Methyl impurity)	0.01	0.029	3	10.1
Impurity-G (Dihydropyridine impurity)	0.004	0.012	3.2	10.2
Salicylic acid impurity	0.001	0.004	3.3	9.5
Esomeprazole Magnesium	0.006	0.021	3.1	10.5

**Tab. 6.** Linearity

**Name**	**Coefficient of correlation (r)**	**Relative response factor**
Impurity-A (Benzimidazole impurity)	0.998	2.85
Impurity-B (Desmethoxy impurity)	0.998	1.10
Impurity-C (Sulphide impurity)	0.997	1.05
Impurity-D (Sulphone impurity)	0.998	0.94
Impurity-E (N-Oxide impurity)	0.998	0.93
Impurity-F (N-Methyl impurity)	0.998	0.81
Impurity-G (Dihydropyridine impurity)	0.998	1.47
Salicylic acid impurity	0.999	1.00
Esomeprazole Magnesium	0.999	1.00

**Tab. 7.** Linearity and Range at LOQ level

**Name**	**Spike level**	**Mean % Recovery**
Impurity-A (Benzimidazole impurity)	At LOQ	91.8
	At 150%	109.9
Impurity-B (Desmethoxy impurity)	At LOQ	105.3
	At 150%	100.6
Impurity-C (Sulphide impurity)	At LOQ	109.0
	At 150%	97.6
Impurity-C (Sulphone impurity)	At LOQ	105.0
	At 150%	97.6
Impurity-E (N-Oxide impurity	At LOQ	97.5
	At 150%	96.3
Impurity-F (N-Methyl impurity)	At LOQ	102.8
	At 150%	99.9
Impurity-G (Dihydropyridine impurity)	At LOQ	106.7
	At 150%	101.7
Salicylic acid impurity	At LOQ	101.6
	At 150%	105.6
Esomeprazole magnesium	At LOQ	101.7
	At 150%	110.1
